# Tau Enhances α-Synuclein Aggregation and Toxicity in Cellular Models of Synucleinopathy

**DOI:** 10.1371/journal.pone.0026609

**Published:** 2011-10-24

**Authors:** Nahuai Badiola, Rita Machado de Oliveira, Federico Herrera, Cristina Guardia-Laguarta, Susana A. Gonçalves, Marta Pera, Marc Suárez-Calvet, Jordi Clarimon, Tiago Fleming Outeiro, Alberto Lleó

**Affiliations:** 1 Instituto de Investigacions Biomediques Sant Pau, Hospital de Sant Pau, Barcelona, Spain; 2 Centro de Investigación Biomédica en Red para enfermedades Neurodegenerativas (CIBERNED), Madrid, Spain; 3 Cell and Molecular Neuroscience Unit, Instituto de Medicina Molecular, Lisbon, Portugal; 4 Faculdade de Medicina da Universidade de Lisboa, Lisboa, Portugal; Thomas Jefferson University, United States of America

## Abstract

**Background:**

The simultaneous accumulation of different misfolded proteins in the central nervous system is a common feature in many neurodegenerative diseases. In most cases, co-occurrence of abnormal deposited proteins is observed in different brain regions and cell populations, but, in some instances, the proteins can be found in the same cellular aggregates. Co-occurrence of tau and α-synuclein (α-syn) aggregates has been described in neurodegenerative disorders with primary deposition of α-syn, such as Parkinson's disease and dementia with Lewy bodies. Although it is known that tau and α-syn have pathological synergistic effects on their mutual fibrillization, the underlying biological effects remain unclear.

**Methodology/Principal Findings:**

We used different cell models of synucleinopathy to investigate the effects of tau on α-syn aggregation. Using confocal microscopy and FRET–based techniques we observed that tau colocalized and interacted with α-syn aggregates. We also found that tau overexpression changed the pattern of α-syn aggregation, reducing the size and increasing the number of aggregates. This shift was accompanied by an increase in the levels of insoluble α-syn. Furthermore, co-transfection of tau increased secreted α-syn and cytotoxicity.

**Conclusions/Significance:**

Our data suggest that tau enhances α-syn aggregation and toxicity and disrupts α-syn inclusion formation. This pathological synergistic effect between tau and α-syn may amplify the deleterious process and spread the damage in neurodegenerative diseases that show co-occurrence of both pathologies.

## Introduction

Synucleinopathies are neurodegenerative disorders characterized by the abnormal deposition of α-synuclein (α-syn) in filamentous intracellular inclusions known as Lewy bodies (LBs). The most common synucleinopathy is Parkinson's Disease (PD), but other neurodegenerative diseases share this pathological feature. These include dementia with Lewy bodies (DLB) and multiple system atrophy [1]. The discovery of mutations in the *SNCA* gene, which encodes α-syn, in familial forms of PD or DLB has provided strong evidence for a central role of α-syn in synucleinopathies [2], [3], [4], [5], [6], [7]. Further support was provided by studies that showed that elevated levels of soluble oligomers of α-syn can be detected in the brain homogenates of patients with PD and DLB [8], [9].

The simultaneous accumulation of different proteins in the central nervous system (CNS) is a common feature in many degenerative diseases. In particular, the deposition of tau and α-syn in the CNS has been described in disorders with primary deposition of α-syn, such as familial and sporadic PD, sporadic DLB, and multiple system atrophy [10], [11], [12], [13], [14], [15], [16], [17]. In DLB, tau-positive LBs are typically restricted to limbic areas and, in most of cases, associated with Aβ deposits [11], [13], [14], [15], [16], [18]. Conversely, α-syn deposition has been identified in some patients with disorders characterized by prominent tau pathology, such as familial and sporadic AD [19], [20], [21], Down syndrome [22], progressive supranuclear palsy [23], Parkinsonism dementia complex of Guam [24], and frontotemporal dementia [25], [26]. In these cases, colocalization of tau and α-syn aggregates is also typically restricted to the amygdala and other limbic areas [27]. Additional data supporting a connection between α-syn and tau comes from genetic studies that link the *MAPT* gene, which encodes tau, with increased risk of sporadic PD [28], [29], [30]. Although the co-occurrence of α-syn and tau pathologies has been reported in many neurodegenerative disorders, the functional consequences of the primary deposited protein on secondary pathology has been investigated very little.

We have recently described a family with four affected members with clinical features of DLB [11]. The most distinctive clinical characteristic of this family was an age of onset in the mid 20 s. Neuropathological examination of the proband also disclosed unusual features, in particular generalized LB pathology and neurofibrillary tangles that colocalized in most of the affected neurons. No amyloid deposits were detected in any brain region. The unusual neuropathological feature in this family together with the observation that coexistence of α-syn and tau pathologies is common in sporadic DLB led us to hypothesize a possible pathological synergistic effect between tau and α-syn. In order to test this hypothesis, in the present article we investigated the effects of tau in various neuronal cell models of α-syn aggregation. We found that tau colocalized with α-syn aggregates in a human cell line and primary neuronal cultures. In addition, overexpression of tau increased the number of α-syn aggregates, the levels of high molecular weight species of α-syn, and enhanced α-syn toxicity.

## Results

### Tau colocalizes with α-syn aggregates

**Figure 1:**
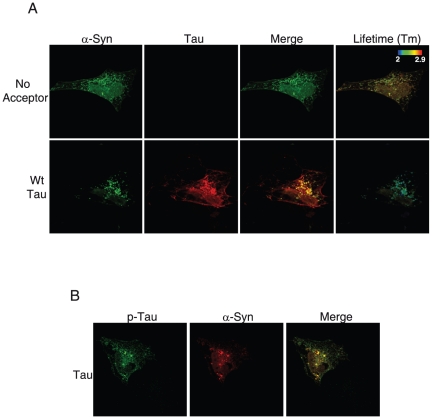
Tau and phospho-tau colocalize and interact with α-syn aggregates in H4 neuroglioma cells. H4 cells were co-transfected with syn-T plus synphilin1-V5 and empty vector or tau. *A*, After 48 h cells were fixed and immunostained for α-syn and tau with Alexa Fluor 488 and Alexa 555-labeled antibodies, respectively. Images were analyzed by confocal microscopy and FLIM. Tau and α-syn colocalize in some aggregates but not in all of them (bottom panels). Fluorescence lifetime values are shown in the pseudocolored image. The closeness between α-syn and tau in some aggregates is reflected by a shorter lifetime of Alexa Fluor 488 in the presence of tau. This is represented as a shift from red-yellow towards the green-blue pseudocolor. *B,* H4 cells were fixed and immunostained for phospho-tau and α-syn with Alexa Fluor 488 and Alexa 555-conjugated antibodies, respectively. Colocalization between phospho-tau and α-syn was observed in some aggregates.

To test the effects of tau on α-syn aggregation we used a well-established model in which co-transfection of α-syn (syn-T) and synphilin1-V5 leads to α-syn aggregation [31]. This model is based on the expression of α-syn tagged with a truncated non-fluorescent form of GFP (syn-T). It has been previously shown that the expression of GFP-tagged tubulin or synaptophysin does not form aggregates [32], suggesting that α-syn is the primary element driving aggregation in the syn-T model. H4 cells were co-transfected with syn-T, synphilin1 and tau and immunostained with antibodies against tau and α-syn. We observed that tau colocalized with α-syn in some but not all aggregates using confocal microscopy (Fig. 1A). As a control, we co-transfected H4 cells with syn-T, synphilin1 and mCherry-tubulin and we did not observe colocalization of α-syn aggregates with tubulin (Fig. S1). Since colocalization has a spatial resolution of >250 nm, we used fluorescence lifetime imaging microscopy (FLIM) to test whether tau and α-syn interact more closely. FLIM is a Förster resonance energy transfer (FRET)-based assay that relies on the principle that when two specific fluorophores are in close proximity (<10 nm), the measured fluorescence lifetime of the donor fluorophore is shortened in proportion to the distance between the fluorophores [33], [34]. In our α-syn inclusion model, we double immunostained with antibodies against α-syn and tau with Alexa Fluor 488 and 555-labeled antibodies, respectively. The donor fluorophore (Alexa Fluor 488, α-syn) had a fluorescence lifetime of ∼2.6 ns in the absence of a FRET acceptor. In some aggregates, the acceptor (Alexa Fluor 555, tau) was in close proximity to the donor, and the lifetime was shortened to ∼2.2 ns. The shortening in fluorescence lifetime is represented in the pseudocolored image by a shift towards the green-blue color (Fig. 1A). In addition, phosphorylated tau also colocalized with α-syn aggregates (Fig. 1B).

**Figure 2:**
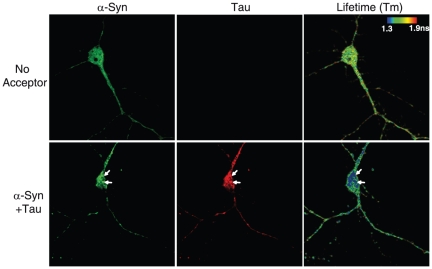
Tau colocalizes and interacts with α-syn aggregates in mouse cortical neurons. Cortical neurons were co-transfected after 7 days in vitro with α-syn-GFP and tau-RFP. Neurons were then fixed and immunostained for α-syn and tau with Alexa Fluor 488 and Alexa Fluor 555-conjugated antibodies, respectively. Images were analyzed by confocal microscopy and FLIM. The close proximity between α-syn and tau is reflected by a shift towards the green-blue color. The shortening in fluorescence lifetime is particularly evident in some aggregates (arrows).

The interaction between tau and α-syn was further confirmed in primary cultured neurons. It has been previously shown that overexpression of α-syn-GFP in hippocampal neurons generates α-syn aggregates [32]. Mouse primary cortical neurons were transfected with α-syn-GFP and tau-RFP and their colocalization was assessed by confocal microscopy. Tau and α-syn colocalized not only in aggregates but also in other parts of the cell (Fig. 2). The interaction was also confirmed using FLIM. A clear decrease in Alexa Fluor 488 lifetime was detected in neurons transfected with α-syn and tau when compared to neurons transfected only with α-syn. The lifetime shortening was particularly evident in the aggregates (Fig. 2). Taken together, these data indicate that tau and phosphorylated tau colocalize and interact with α-syn in aggregates.

### Overexpression of tau shifts α-syn aggregation pattern

**Figure 3:**
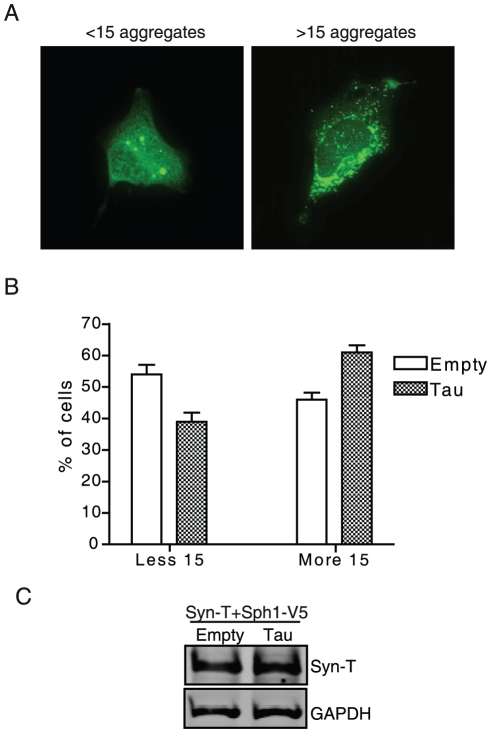
Overexpression of tau shifts the pattern of α-syn aggregation. H4 cells were co-transfected with syn-T plus synphilin1-V5 and empty vector or tau. Co-transfection with tau in this model led to an increase in the number of inclusions and a reduction in their size. *A*, Cells were classified in two groups: cells with less than 15 aggregates and cells with more than 15 aggregates. *B*, Co-transfection of cells with tau increased the percentage of cells with >15 aggregates as compared with those transfected with empty vector (n = 250 cells). Results are mean ± SEM of 4 independent experiments. *C*, Total levels of syn-T were measured by western blot. GAPDH was used as a loading control. A representative experiment is shown (n = 4 independent experiments).

Next, we asked whether the observed colocalization and interaction between tau and α-syn would alter the aggregation pattern of α-syn. Co-transfection with tau resulted in an increase in the number of α-syn inclusions per cell and a reduction in their size. To quantify this shift in the aggregation pattern we classified cells in an unbiased manner into two groups: cells with less than 15 aggregates and cells with more than 15 aggregates (Fig. 3A). We detected a ∼25% increase of cells with more than 15 aggregates when tau was co-transfected (Fig. 3B). These data suggest that tau shifts the α-syn aggregation pattern and favours the formation of more and smaller aggregates. To test whether the increase of aggregates was due to redistribution of insoluble α-syn or to an increase of total α-syn levels, we measured the levels of α-syn in the absence or presence of tau. After quantification, we found no difference in total syn-T levels when cells were co-transfected with tau (Fig. 3C).

### Tau increases insoluble high molecular weight (HMW) species of α-syn and promotes secretion of α-syn

**Figure 4:**
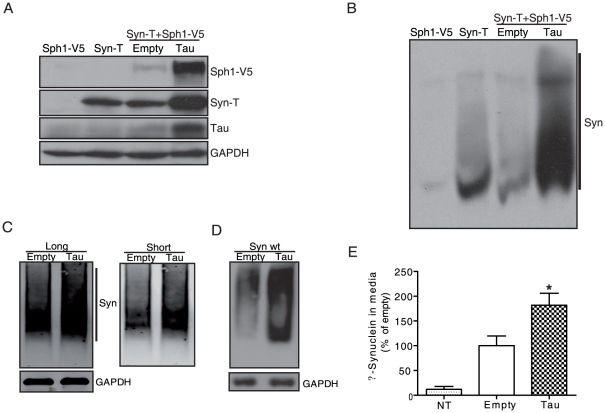
Tau increases insoluble α-syn, high molecular weight species and extracellular α-syn levels. H4 cells were co-transfected with syn-T plus syphilin1-V5 and empty vector or tau. After 48 h cells were lysed and the insoluble fraction was resolved in SDS-PAGE (*A*) or native-PAGE (*B*) gels. When tau was co-transfected, we observed an increase in the levels of insoluble α-syn and HMW species of α-syn. GAPDH was used as a loading control. A representative experiment is shown (n = 4 independent experiments). *C,* Stable H4 cells expressing α-syn constructs (GN-link-aSyn and aSyn-GC) were transfected with either empty vector or tau. After 36 h, cells were lysed and the sample was run under non-denaturing conditions. An increase in the smear was observed in cells co-transfected with tau. GAPDH was used as a loading control. A representative experiment is shown (n = 4 independent experiments). *D*, H4 cells were co-transfected with wt α-syn plus empty vector or tau. After 48 h, cells were lysed and the sample was run under non-denaturing conditions. An increase of α-syn HMW species was observed in cells co-transfected with tau. GAPDH was used as a loading control. A representative experiment is shown (n = 3 independent experiments). *E*, H4 cells were co-transfected with syn-T plus synphilin1-V5 and either empty vector or tau. Non-transfected cells (NT) were used as a control. After 48 h, conditioned medium was collected and the levels of α-syn in media were measured by ELISA. Levels of extracellular α-syn were higher in cells transfected with syn-T plus synphilin1-V5, and co-transfection with tau further increased α-syn extracellular levels. Data are mean ± SEM of 4 independent experiments performed in duplicate. * p<0.05 compared to empty vector.

Next, we tested whether the observed increase in the number of aggregates in the presence of tau could modify insoluble α-syn species in our inclusion model. We analyzed the NP40-insoluble fraction by SDS-PAGE in H4 cells co-transfected with empty vector or tau. In cells co-transfected with tau, there was an increase in both syn-T and synphilin1-V5 protein levels, indicating that tau enhances the accumulation of insoluble α-syn (Fig. 4A). Moreover, native PAGE of the NP40-insoluble fraction revealed an increase in HMW species of α-syn when tau was co-transfected (Fig. 4B). To further confirm the enhancement of α-syn aggregation by tau, we performed the same experiments in another cell model. By using a protein-fragment complementation assay, it has been shown that transfection of H4 cells with non-fluorescent fragments of GFP fused to α-syn (GN-link-aSyn and aSyn-GC) can generate HMW species of α-syn [35]. These HMW species can be analyzed by either fluorescence microscopy or by western blot, where the smear reflects a wide range of α-syn oligomeric species [35]. Here, H4 cells stably expressing GN-link-aSyn and aSyn-GC were transfected with either tau or an empty vector. Native PAGE of total cell lysates was performed and a smear indicative of HMW species of α-syn was detected. Under these conditions, an increase in the smear was observed in cells transfected with tau (Fig. 4C). To rule out the possibility that the observed effects of tau on α-syn aggregation were mediated by the presence of tagged α-syn, we repeated our experiments using untagged α-syn. In H4 cells transfected with wt α-syn and tau, an increase in the smear was also observed when compared with cells transfected with wt α-syn (Fig. 4D). These results suggest that tau enhances the formation of insoluble HMW species of α-syn in different neuronal cell models.

Different studies have indicated that α-syn can be secreted from neuronal cells [36], [37], [38], [39], suggesting a mechanism of pathological propagation in synucleinopathies. We therefore asked whether tau could promote the secretion of α-syn in our α-syn inclusion model. We co-transfected cells with empty vector or tau and measured the levels of α-syn in the conditioned medium. We found that co-transfection with tau increased the levels of secreted α-syn by 80% (Fig. 4E).

### Tau enhances α-syn toxicity

**Figure 5:**
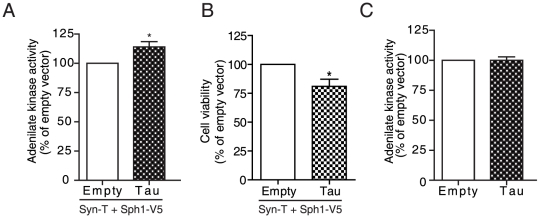
Tau increases α-syn cytotoxicity. *A*, H4 cells were co-transfected with syn-T plus synphilin1-V5 and either empty vector or tau. Conditioned medium was collected 48 hours after transfection, and toxicity was determined by measuring the activity of adenylate kinase. Co-transfection of cells with tau in the α-syn inclusion model increased the levels of adenylate kinase in the medium, indicative of an increase in cytotoxicity. Results are mean ± SEM of 7 independent experiments performed in triplicate. *p<0.05 compared to empty vector. *B*, H4 cells were co-transfected with syn-T plus synphilin1-V5 and either empty vector or tau. After 48 h cell viability was determined by MTT assay. Data are mean ± SEM of 3 independent experiments performed in quadruplicate. *p<0.05 compared to empty vector. *C*, H4 cells were transfected with empty vector or tau. Conditioned medium was collected 48 hours after transfection, and adenylate kinase activity was determined. Transfection of tau does not induce significant toxicity in the absence of α-syn aggregates. Results are mean ± SEM of 5 independent experiments performed in triplicate. * p<0.05 compared to empty vector.

Finally, we asked whether the increase in α-syn aggregates in the presence of tau could affect cell viability. We used the release of adenylate kinase to the culture medium as a measure of cytotoxicity, which has been demonstrated to be a sensitive and consistent assay in α-syn cell models [35], [40]. We observed a significant increase in cytotoxicity in cells co-transfected with tau compared to cells transfected with an empty vector (Fig. 5A). These results were confirmed using the commonly used MTT cell viability assay (Fig. 5B). Cells transfected with tau showed a decrease in cell viability when compared to cells transfected with an empty vector. To rule out the possibility that the toxicity was merely due to tau overexpression, we transfected H4 cells with tau vector in the absence of α-syn. As shown in Fig. 5C, there was no increase in cytotoxicity under these conditions. Taken together, these data suggest that tau enhances α-syn cytotoxicity.

## Discussion

In this study, we showed that tau colocalized and interacted with α-syn aggregates in H4 cells and primary neuronal cultures. This interaction is associated with more α-syn aggregates, HMW species and enhanced toxicity. Thus our results support the notion that tau is not simply a bystander, but rather enhances the pathological aggregation of α-syn.

α-syn is a presynaptic protein localized mainly in axon terminals that plays a role in synaptic function [41]. Tau is a microtubule-associated protein localized along the axon that stabilizes microtubules [42], [43] and is involved in cellular trafficking and axonal transport [44]. Both are highly expressed in the CNS, are synthesized as native unfolded proteins and have the propensity to form pathological insoluble intracellular aggregates in the CNS in different neurodegenerative diseases. Few studies have investigated the interaction between these two proteins. Initial studies identified soluble tau as a ligand for α-syn by affinity chromatography and direct binding studies [45]. α-syn has also been shown to stimulate tau phosphorylation at different serine residues in different cellular and animal models [45], [46], [47], [48]. This functional link could be relevant for many neurodegenerative diseases in which α-syn and tau co-aggregate, since an increase in hyperphosphorylated tau is one of the key features in the brains of all tauopathies and has been shown to reduce binding of tau to microtubules [49]. Other studies have also suggested a pathological synergistic effect between α-syn and tau. α-syn can aggregate *in vitro*, and this is greatly enhanced by co-incubation with tau in a concentration-dependent manner [14]. Interestingly, the effects were specific for tau because co-incubation with Aβ did not enhance α-syn polymerization [14]. More recently, data obtained from a novel cellular model demonstrated that few amounts of fibrillized α-syn seeds are able to induce intracellular massive tau aggregation [50]. These tau aggregates were hyperphosphorylated and occupied almost all the soma, sharing similar characteristics with neurofibrillary tangles. These data support the idea that cross-seeding of pathologic proteins can occur in some neurodegenerative diseases. Transgenic animal studies also support a synergistic effect between Aβ, tau and α-syn pathologies [51]. In particular, a transgenic mouse model that develops Aβ, tau and α-syn pathologies exhibits accelerated cognitive decline with enhancement of all three pathologies. Although it is difficult to determine whether the increase in α-syn and tau pathologies in this model is also mediated by Aβ, the results clearly support the notion that Aβ, tau and α-syn promote the aggregation of each other. In this study, we confirm and extend these observations in various neuronal models of synucleinopathy. The tau-induced increase in the number of aggregates and HMW species of α-syn observed in our study provides evidence that tau synergistically affects the polymerization of α-syn. In particular, tau increased the levels of insoluble syn-T rather than affecting total levels. A possible explanation for the increase in the insoluble syn-T fraction is the ability of tau to inhibit histone deacetylase 6 (HDAC6), a key component of the aggresome complex [52]. This inhibitory effect of tau on HDAC6 could affect the transport of small aggregates to aggresomes or the assembly into larger aggregates. In addition, our observation that co-expression of tau leads to smaller α-syn inclusions and enhanced toxicity may suggest that microaggregates are one of the key factors that mediate toxicity in our model. This is consistent with studies that show a massive presence of small aggregates in presynaptic terminals associated with loss of dendritic spines in the brains of patients with DLB [53]. Taken together, these results support the idea that microaggregates, rather than LBs, likely represent the pathogenic component that drive neurodegeneration in synucleinopathies. The increase in α-syn HMW species in the presence of tau in our study also provides evidence for a central role of soluble oligomers in DLB and other synucleinopathies. It is tempting to speculate that, in the presence of tau, these microaggregates may drive dendritic and synaptic damage by slowly releasing oligomeric forms of α-syn.

Different studies have identified that monomeric and oligomeric α-syn are secreted to the extracellular medium from neuronal cells via exocytosis [36], [37], [38], [39]. These findings provide strong evidence for direct cell-to-cell propagation of α-syn, similar to that observed in prion diseases [54]. Here, we detected an increase in α-syn secretion when tau was overexpressed. Although we cannot completely rule out that the increase in extracellular α-syn levels in the presence of tau is due to membrane leakage, the large increase observed compared to the slight toxicity argues against this possibility. Instead, this suggests that α-syn secretion might be triggered by the toxic properties of overexpressed α-syn and that tau specifically enhances α-syn secretion. This tau-enhanced secretion of α-syn may be relevant in cases with a co-occurrence of α-syn and tau pathologies, such as our early-onset DLB family [11]. Together, our data reinforce the notion that synergistic effects between α-syn and tau may be a relevant disease component that enhances the pathological cascade and spreads the damage in neurodegenerative diseases that show co-occurrence of both pathologies.

## Materials and Methods

### Plasmids, Cell lines and Transfections

A construct encoding for the human wild-type α-syn with a C-terminal tag corresponding to a truncated fragment of GFP (referred to as syn-T) was used in combination with a construct encoding synphilin1 (tagged with a V5 tag) as a model of α-syn aggregation [31], [32]. Alternatively, constructs encoding for the human wild-type untagged or GFP-tagged α-syn were used alone [31]. The construct encoding for the longest isoform (2N4R) of human wild-type tau (referred to as tau) was a kind gift from Jesus Avila (Centro de Biología Molecular, Madrid, Spain), and the construct encoding for the human wild-type tau tagged with RFP was a kind gift from Rohan de Silva (UCL Institute of Neurology, London, UK). Where indicated, cells were co-transfected with a construct encoding for α-tubulin tagged with mCherry. The plasmid was a kind gift from Domingos Henrique (Instituto de Medicina Molecular, Lisbon, Portugal).

Human neuroglioma (H4) cells and primary neuronal cultures were used in this work. H4 cells were maintained in OPTI-MEM with 10% FBS at 37°C and plated onto four-well chamber slides (Labtek, Nalge-Nunc, Naperville, IL, USA) or 10 cm dishes 24 h prior to transfection. H4 cells were transfected with equimolar amounts of the constructs encoding syn-T and synphilin1-V5 and either empty vector or a construct encoding for tau. The percentage of cells transfected with the three plasmids was ∼5–10%. H4 cells stably expressing GN-link-αSyn and αSyn-GC were transfected with either empty vector or a construct encoding for tau.

Mouse cortical neurons were prepared as previously described [33]. Neurons were cultured in Neurobasal medium containing B27 supplement (Invitrogen, Carlsbad, CA, USA). After 7 days *in vitro*, cells were transfected with α-syn-GFP and tau-RFP constructs and immunostained.

H4 cells and mouse cortical neurons were transiently transfected with Fugene 6 (Roche Diagnostics, Mannheim, Germany) and Lipofectamine 2000 (Invitrogen, Carlsbad, CA, USA) reagents, respectively, according to the manufacturer's instructions.

### Cytotoxicity Assays

Cells were plated onto 6-well plates at 200.000 cells/ml density 24h before transfection. Conditioned medium was collected 48 h after transfection, centrifuged at 10000xg at 4°C for 5 min to remove cells in suspension, and the activity of adenylate kinase was measured using ToxiLight^TM^ (Cambrex, Walkersville, MD, USA), according to the manufacturer's protocol. Cell viability was also monitored by the colorimetric 3-(4,5-dimethylthiazol-2-yl)-2,5-diphenyl tetrazolium bromide (MTT) assay as previously described [55]. MTT (0.5 mg/ml) was added to cultures and incubated for 2h at 37°C. The reaction media were then aspirated and DMSO was used to solubilize the blue formazan product prior its quantification at 570 nm (ELx-800 Absorbance microplate reader, BioTek). Results were expressed as the percentage of viable cells.

### Immunocytochemistry and antibodies

Cells were fixed and immunostained 48 h after transfection as described elsewhere [56]. Briefly, paraformaldehyde-fixed cells were incubated with primary antibodies overnight at 4°C. Cells were washed three times with TBS-Tween 0.1%, and incubated with Alexa Fluor 488- or 555-labeled secondary antibodies (1:500; Invitrogen, Carlsbad, CA, USA) for 1 hour at room temperature. Cells were then washed three times with TBS-Tween 0.1% and kept at 4°C in TBS until visualization. We used the following primary antibodies: syn-1 (1:1000, BD Transduction Labs, San Jose, CA, USA), α-syn (1:100, Cell Signalling Technology, Danvers, MA, USA), Tau-13 (1:200, MBL, Nagoya, Japan), Tau HT7 (1:100, Innogenetics, Ghent, Belgium), ser396/404 phospho-tau (PHF1, a kind gift from Peter Davies, Albert Einstein College of Medicine, NY).

### Confocal microscopy and Fluorescence Lifetime Imaging Microscopy (FLIM)

Confocal microscopy and FLIM was performed using a Leica inverted fluorescent confocal microscope (Leica TCD SP5-AOBS, Wetzlar, Germany). FLIM has been described previously as a novel technique for the analysis of protein proximity [34], [57]. The technique is based on the observation that fluorescence lifetimes of a donor fluorophore shorten in the presence of a FRET acceptor in close proximity (<10 nm). The decrease in lifetime is proportional to the distance between the fluorophores at R^6^. The Leica SP5 confocal microscope used to perform FRET/FLIM experiments was equipped with three pulsed lasers (405, 470, 640 nm) and time-correlated single photon counting detectors. The hardware/software package allows the measurement of fluorescence lifetimes on a pixel-by-pixel basis with high spatial resolution. Donor fluorophore (Alexa Fluor 488) lifetimes were fitted to two exponential decay curves as described [34], [57]. For α-syn-tau FRET/FLIM experiments, cells were fixed and double-immunostained for α-syn and tau as described above. As a positive control, cells were immunostained against α-syn and then incubated with equimolar concentrations of Alexa Fluor 488 and Alexa 555-labeled secondary antibodies (Invitrogen, Carlsbad, CA, USA; Fig. S2). All samples were compared to a negative control in which the donor (Alexa Fluor 488) fluorescence lifetime was measured in the absence of the acceptor (no FRET ∼2500 ps).

### Quantification of α-syn inclusions

Cells were analyzed by two independent observers blinded to the experimental conditions. Ten 20x fields were assessed for each well and two wells were assessed for each condition and experiment. Each field contained 1–10 transfected cells and 80–100 cells were assessed for each experiment. A total of four independent experiments were performed for each condition. Transfected cells were detected and scored based on the α-syn aggregation pattern. Cells were classified into two groups: cells with less than 15 aggregates and cells with more than 15 aggregates. Results were expressed as a percentage of the total number of transfected cells.

### Detergent-solubility fractionation

Cells were lysed in 1% NP-40 buffer and lysates were incubated for 1 hour on ice and centrifuged at 15,000x*g* for 60 min at 4°C. The supernatant was collected and designated NP-40-soluble fraction, and the pellet was resuspended in the same lysis buffer, sonicated twice for 10 s and collected as the NP-40-insoluble fraction.

### Western Blot

Lysate protein concentration was determined using a Bradford protein assay. Each cell lysate (20–40 µg) was electrophoresed in 10% Tris-glycine gels for Western blot analysis. SDS-PAGE was performed with SDS-containing running and sample loading buffers, whereas native-PAGE was performed with 4–15% TGX^TM^ gels (Biorad, Richmond, CA, USA) using SDS-free running and sample loading buffers. The immunoblotting was performed overnight at 4°C with the following primary antibodies: α-syn (Syn-1, 1:1,000, BD Transduction Laboratories, San Jose, CA, USA), anti-V5 (1:1000, Abcam, Cambridge, UK), GAPDH (1:10,000, Millipore, Bedford, MA, USA), or Tau HT7 (1:500, Innogenetics, Ghent, Belgium). The immunoblotting was followed by detection with a HRP-conjugated secondary antibody and enhanced chemiluminescence substrate (Millipore, Bedford, MA, USA) or with an infrared fluorescent-labelled secondary antibody (IRDye, Rockland Immunochemicals, Gilbertsville, PA). Bands on films were quantified using Odyssey software (LI-COR, Lincoln, NE, USA).

### Human soluble α-syn ELISA

Cells were plated onto 6-well plates at 200.000 cells/ml density 24 h before transfection. Conditioned medium was collected 48 h after transfection, centrifuged at 10000xg at 4°C for 5 min to remove cells in suspension, and the concentration of α-syn was quantified using an ELISA kit (Invitrogen, Carlsbad, CA, USA), according to the manufacturer's protocol. Briefly, samples are loaded into wells coated with a specific α-syn monoclonal antibody. After incubation, detection α-syn rabbit polyclonal antibody is loaded into the wells. After washing, a HRP-conjugated anti-rabbit IgG (anti-rabbit IgG HRP) is added. After incubation and washing, a substrate solution is added. Absorbance is then read at 450 nm. In this assay the intensity of this colored product is directly proportional to the concentration of α-syn present in the original specimen.

### Statistical Analysis

Data are shown as mean ± standard error (SEM) of at least 3 independent experiments. Statistical significance was determined by one-way analysis of variance (ANOVA) followed by Tukey's multiple comparison test. The level of significance was set at 5% (α = 0.05).

## Supporting Information

Figure S1 **Tubulin does not colocalize with α-syn aggregates.** H4 cells were co-transfected with syn-T plus synphilin1-V5 and mCherry-tubulin. After 48 h cells were fixed and immunostained for α-syn with a primary and an Alexa Fluor 488-labeled secondary antibodies. Images were analyzed by confocal microscopy and no colocalization between α-syn and tubulin was observed.
(EPS)

Figure S2 **Positive control for the** **FLIM assay.** H4 cells were co-transfected with syn-T and synphilin1-V5, fixed and incubated with a primary antibody against α-syn. Cells were then washed and incubated with equimolar concentrations of Alexa Fluor 488 and Alexa Fluor 555-labeled secondary antibodies. As expected, we observed wide colocalization and the donor fluorescence lifetime was significantly shortened as reflected by the blue pixels in the pseudocolored lifetime image. Photobleaching of the FRET acceptor (square) led to an almost complete loss of the FRET signal with an increase in the lifetime of the donor fluorophore. This is reflected by a shift towards green in the pseudocolored lifetime image.
(EPS)
